# Effects of acupuncture on improving sleep quality and the risk of emotional maladjustment of breast cancer patients: a systematic review and meta-analysis

**DOI:** 10.3389/fonc.2025.1617818

**Published:** 2025-06-26

**Authors:** Yi Yang, Hong Wang, Zai Zhao Chen, Qi Rui Teng

**Affiliations:** Jiang Xi University of Traditional Chinese Medicine, Nanchang, China

**Keywords:** acupuncture, breast cancer, risk of emotional maladjustment, meta-analysis, sleep quality

## Abstract

**Background:**

Acupuncture is highly controversial in enhancing the quality of sleep and the risk of emotional maladjustment in breast cancer patients. Through a comprehensive meta-analytic approach, this study intends to investigate the effects of acupuncture on enhancing sleep quality and the risk of emotional maladjustment in patients diagnosed with breast carcinoma.

**Methods:**

Search tactics aligned with Cochrane Collaboration recommendations. Systematic electronic searches were conducted in April 2025 across several data storage, including PubMed, Embase, Web of Science, the Cochrane Library, and Scopus. The reference lists of review essay were manually searched to refine potentially eligible studies. A pair of researchers independently performed a comprehensive literature review, conducted data extraction, and assessed the risk of bias. Outcome analysis included the quality of sleep and the risk of emotional maladjustment. For continuous measures, the pooled weighted mean difference (MD) and 95% confidence interval will be estimated. The meta-analysis was conducted using the Review Manager 5.3 and Stata 16.0 statistical software platforms.

**Result:**

A total of 11 studies involving 1116 participants were included. Statistically significant differences were found between acupuncture and control group in Pittsburgh Sleep Quality Index (PSQI) (MD = -1.38, 95%CI = -2.45 to -0.31), Insomnia Severity Index (ISI) (MD = -1.11, 95%CI = -3.68 to 1.45), Brief Fatigue Scale (BFI) score (MD = -2.30, 95%CI = -5.62 to -1.03). Acupuncture has been shown to Boost sleep quality and reducing the risk of emotional maladjustment. Specifically, it is manifested in: Hospital Anxiety and Depression Scale (HADS-A) (MD = -1.24, 95%CI = -2.41 to -0.35) and Hospital Anxiety and Depression Scale (HADS-D) (MD = -1.18, 95%CI = -2.15 to -0.20).

**Conclusion:**

In comparison to other standard treatment modalities, acupuncture exhibits a notable distinction in boosting the sleep quality and the risk of emotional maladjustment of breast cancer patients. However, Clinicians should exercise caution when applying these results, as the available evidence suffers from methodological biases and quality limitations.

## Introduction

Breast cancer is the most prevalent form of cancer among women globally, predominantly affecting those aged between 41 and 60 years. In 2014, the National Cancer Institute reported an incidence rate of 5,609 cases per 100,000 women (1). In a document, Global Cancer Statistics 2020, Breast cancer has now overtaken lung cancer. It has 2.26 million cases (2). In female patients, Breast cancer has become the leading malignant cancer in women. With the rising standard of living, the survival rates for breast cancer are improving. His 5-year survival rate is 90% (3). Sleep quality is most prominently observed in the progression of breast cancer patients (4). A systematic review demonstrated that 62% of quality-of-life deterioration in breast cancer cases was attributable to sleep disturbances (5). In the lives of patients with breast cancer, disturbances in sleep significantly impact their life (6). The incidence of symptoms associated with breast cancer, including fatigue, anxiety, and depression, is notably high. These symptoms significantly increase the risk of emotional dysregulation (7). Therefore, sleep disorders and the risk of emotional maladjustment in breast cancer patients have increasingly attracted attention in clinical and research fields.

There are both pharmacological and non-pharmacological approaches to improving symptom of individuals with breast cancer. These methods can cause tolerance of the drug. During the treatment process of breast cancer, there is this interaction between anti-cancer drugs and sleep medications (8). Therefore, people are looking for various ways to treat sleep problems caused by breast neoplasm and boost their quality of life (9). Exercise (10), acupuncture (11), tai chi (12), yoga exercises (13). Of these methods, acupuncture works best. Acupuncture has a long history as a means of treatment, and acupuncture as a treatment has received a lot of popularity both at home and abroad (14). Acupuncture includes acupuncture and moxibustion. Acupuncture is by piercing small needles into the corresponding acupuncture points to make them “qi” (15). Acupuncture improves the body’s immune function (16). Moxibustion is a method of moxibustion that achieves a therapeutic effect through thermal stimulation of acupuncture points and acupuncture and moxibustion can be used as a treatment method that can be used in conjunction with each other, such as the combination of acupuncture and moxibustion (17). Currently, research has been conducted on the enhancement of quality of life in breast neoplasm treatment through acupuncture. Some research has indicated that acupuncture may enhance the life in breast cancer patients by promoting better sleep quality (18). Acupuncture may enhance sleep quality while concurrently alleviating distress associated with psychiatric symptoms (19). Acupuncture has been shown to be safe for sleep quality (20, 21). However, relevant studies have indicated that the clinical efficacy of acupuncture interventions for cancer-related insomnia and the risk of emotional maladjustment still requires further evidence from randomized controlled trials and systematic reviews to support its validity (22). It cannot be definitively stated that acupuncture is demonstrates efficacy in managing insomnia and emotional maladjustment associated with cancer (23).

Therefore, we conducted this meta-analysis to reevaluate the impact of acupuncture intervention on improving sleep quality and risk of emotional maladjustment in breast cancer patients. This analysis incorporates recent high-quality randomized controlled trials (RCTs) that were not included in previous studies.

## Methods

Because this research is a systematic review and meta-analysis of published studies, ethical approval is not necessary. According to the guidelines established by the Preferred Reporting Items for Systematic Reviews and Meta-Analyses (PRISMA), we conducted a meta-analysis (24), and the guidelines for Assessing the Methodological Quality of Systematic Reviews (AMSTAR) (25). The International Prospective Register of Systematic Reviews has registered this review under CRD420251025827. For details, please refer to attachment 1 of the attached upload materials.

### Search strategy

Search tactics aligned with Cochrane Collaboration recommendations. Systematic electronic searches were conducted in April 2025 across several databases, including PubMed, Embase, Web of Science, the Cochrane Library, and Scopus. Randomized controlled trials were undertaken to investigate the outcome of acupuncture on sleep quality and the risk of emotional maladjustment in breast carcinoma patients. To enhance the comprehensiveness of our review, we also conducted a manual search of the references cited in the included studies. We constructed the search formula by combining Medical Subject Headings (MeSH) with non-MeSH terms (free text). The specific keywords included “acupuncture therapy”, “breast neoplasms”, and “randomized controlled trial”. Comprehensive strategies for detailed keyword searches are obtainable in Multimedia Appendix 1.

### Selection and exclusion criteria

To reduce bias, the studies that aligned with the search strategy were carefully examined and included based on the following criteria: (1) Population: Insomnia patients among breast cancer survivors; (2) Intervention: acupuncture or acupressure; (3) Comparison: No acupuncture treatment or acupressure was performed; (4) Outcome measures: sleep quality and risk of emotional maladjustment; (5) Study design: a randomized controlled trial.

Exclusion criteria: (a) Studies including Non-breast cancer patients; (b) Cases, practice guides, test protocols or reviews; (c) Experimental data are incomplete; (d) There are no full-text or non-RCTs.

### Data extraction

The data extraction was conducted independently by two authors, and any Discrepancies in opinions were resolved by discussion with the third author to establish consensus. The extraction of information was conducted systematically, encompassing various elements such as the first author name, publication year, country of origin, demographic age ranges, the number of experimental and control groups, diagnostic criteria utilized, interventions applied, observed clinical outcomes, and any additional pertinent information.

### Risk of bias assessment

Utilizing the Cochrane Risk of Bias tool, we analyzed the risk of bias in randomized controlled trials (RCTs) as high, low, or unclear. The components involved in bias assessment included, generation of random sequences and concealment of distribution, the implementation of a blind method for both participants, blinding of outcome assessments, selective reporting, and the management of incomplete outcome data (26). If the two authors do not reach a consensus in the process of assessing the risk, a third reviewer will resolve the assessment differences.

### Quality of evidence

According to the Grading of Recommendations Assessment, Development and Evaluation (GRADE) system, the strength of evidence for each research outcome is assessed and classified into quality ratings. There were varying levels of evidence, including very low, low, moderate, and high.

### Data synthesis

The research performed a meta-analysis utilizing the RevMan 5.3 software developed by the Cochrane Collaboration. For continuous data, mean differences (MD) along with their corresponding 95% confidence intervals (CI) were employed for statistical analysis. Researchers measure the odds ratio (OR) along with its 95% confidence interval (95% CI) utilizing the primary model. Heterogeneity was measured by P-values and I² statistics. When P > 0.10 and I² < 50%, it can be determined that there is no significant heterogeneity among the data sets; therefore, a fixed effects model (FEM) is employed. Conversely, when P < 0.10 and I² > 50%, which indicates significant heterogeneity, the random-effects model (REM) was utilized. In instances characterized by significant heterogeneity, we conducted sensitivity analyses or subgroup analyses to investigate the underlying sources of this heterogeneity. According to the origin of the country, it is divided into 2 subgroups: China and Unite States. According to the interventions, it is divided into 2 subgroups: acupuncture and acupressure. According to the Course of intervention, it is divided into 2 subgroups: third week and sixth week. Furthermore, when the number of included studies exceeded 10, funnel were created to identify possible publication bias in the literature reviewed.

## Results

### Study selection and characteristics

**Figure 1:**
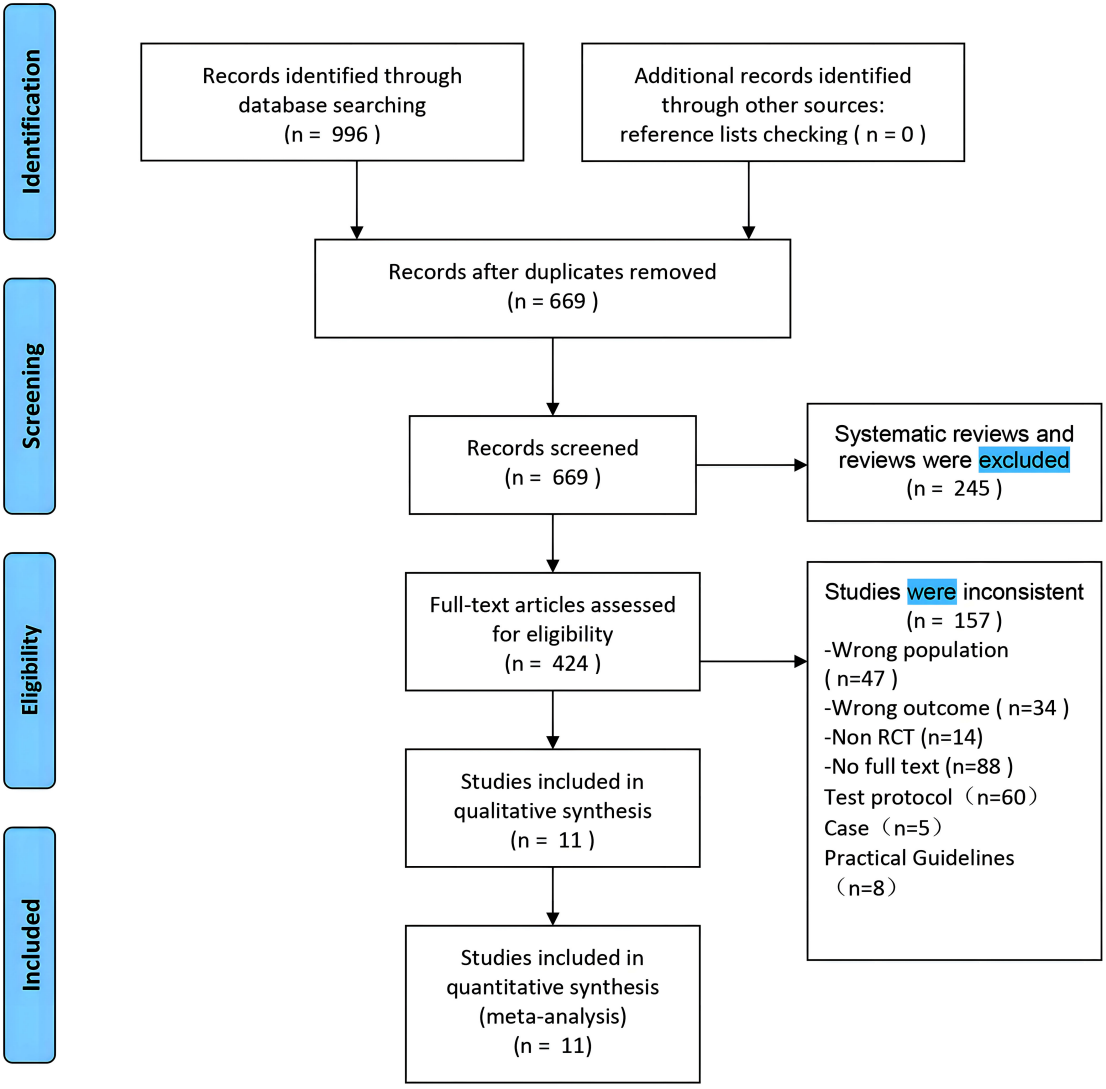
Flowchart of the process that studies identification, screening, inclusion and exclusion.

Using the search strategy, 996 articles were retrieved from the database. After removing duplicates (669 articles remained), we excluded the remaining articles: reviews, non-RCTs, no full text, trial protocols, case reports, practice guidelines, and study populations. This process resulted in 11 randomized controlled trials articles included in the meta-analysis (27–37). The process of retrieval and selection is illustrated in **Figure 1**. The characteristics of these literatures are concluded in **Table 1**. The study included a total of 836 women with breast cancer sleep disorders with an average age of about 50 years. The participants were effectively divided into 2 groups, acupuncture (n = 436) and non-acupuncture (n = 400). Regarding outcomes, Eleven studies provided Patient Pittsburgh Sleep Quality Index (PSQI) (27–37), two studies reported the Change in score of Insomnia Severity Index (ISI) (28, 32), three studies investigated the Brief Fatigue Scale (BFI) score (27, 28, 37), five studies investigated the Hospital Anxiety and Depression Scale (HADS-A) (28, 32, 33, 36, 37), five studies investigated the Hospital Anxiety and Depression Scale (HADS-D) (28, 32, 33, 36, 37).

**Table 1:** Summarizes the characteristics of the included studies in the meta-analysis.

Reference	Average age	Diagnostic criteria	Sample size (T/C)	Intervention therapy	Interventions (T/C)	Outcome measures
Maoetal (27) (China) 2024	59.4 years	BC	Experimental(n=56) control (n=28)	2times/week;6week	Infrared laser moxibustion	PSQI BFI
Zhang et al (28) (China) 2024	52.2years	BC	Experimental(n=69) control (n=69)	2times/week;6week 3times/week;7-18week	acupuncture	PSQI BFI HADS-A HADS-D ISI
Liuetal (29) (China) 2022	51.41years	BC	Experimental(n=36) control (n=34)	1times/week;8week	Acupressure points	PSQI
Wangetal (30) (China) 2022	38.4years	BC	Experimental(n=33) control (n=33)	2times/week;3week	Acupressure points	PSQI HADS
Nourizadehetal (31) (United States) 2022	53.47years	BC	Experimental(n=34) control (n=34)	3times/week;8week	Auriculus compression	PSQI
Zhangetal (32) (China) 2021	55.73years	BC	Experimental(n=26) control (n=26)	2times/week;6week	Ear needles	PSQI HADS-A HADS-D ISI
Hoextermannet al (33) (United States) 2021	52.6years	BC	Experimental(n=15) control (n=15)	-;29week	acupuncture	PSQI HADS-A HADS-D
Garlandetal (34) (United States) 2017	51.7years	BC	Experimental(n=30) control (n=28)	2times/week;2week;(1times/week;persistent6week)	Electroacupuncture	PSQI
Zicketal (35) (United States) 2016	-	BC	Experimental(n=90) control (n=86)	1times/week;6week	Acupressure points	PSQI
Baoetal (36) (United States) 2014	-	BC	Experimental(n=23) control (n=24)	8times/week;8week	acupuncture	PSQI HADS-A HADS-D
Maoetal (37) (United States) 2014	59.7years	BC	Experimental(n=22) control (n=23)	2times/week;2week;(1times/week;persistent6week)	ElectroAcupressure	PSQI BFI HADS-A HADS-D

BC, breast cancer; PSQI, Pittsburgh Sleep Quality Index for Patients; HADS, Hospital Anxiety and Depression Scale; ISI, Insomnia Severity Index (ISI) measure.

### Study quality and risk of bias

**Figure 2:**
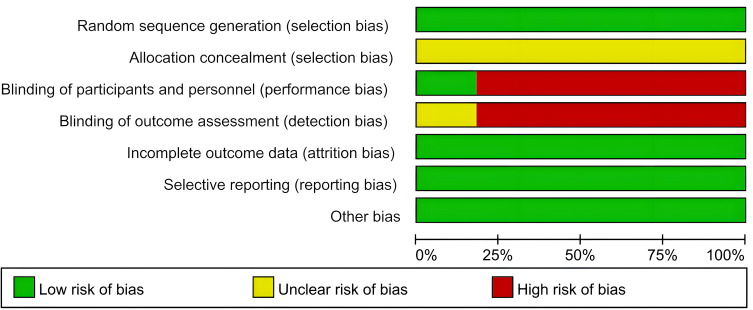
Risk of bias graph.

**Figure 3:**
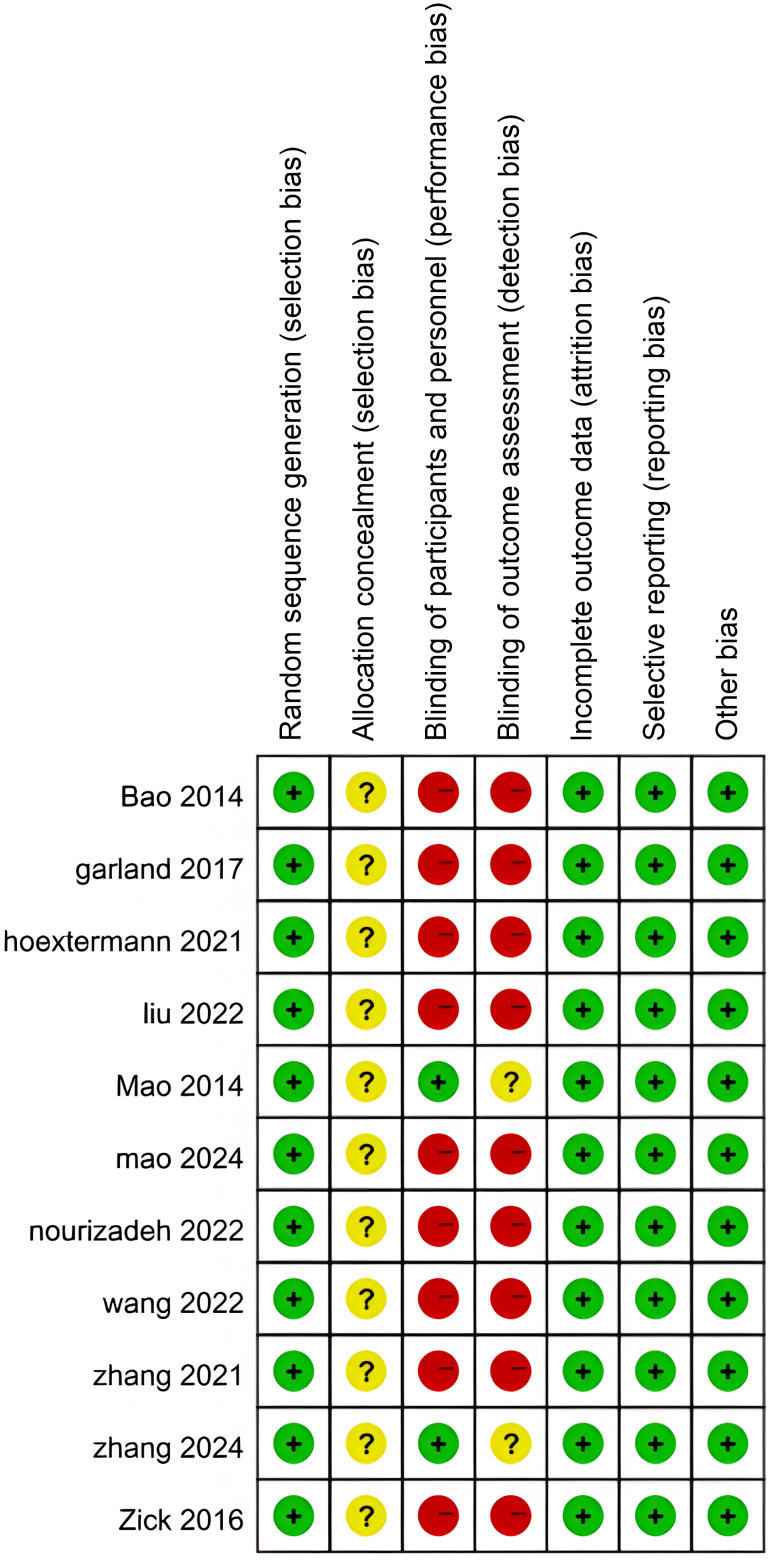
Risk of bias summary.

The quality of the trials included in this review was assessed using the Cochrane tool. Detailed results can be found in **Figures 2** and **3**. In 11 studies, the description of proper random sequence generation was provided (27–37). 2 trails provided adequate blind (28, 37). All studies followed up data and the data were complete, and Detailed results are shown in **Table 2**.

**Table 2:** GRADE assessment.

Outcomes	Study design	Risk of bias	Inconsistency	Indirectness	Imprecision	Other considerations	No. of participants		Absolute effect (95% CI)	Quality
							Acupuncture	Control		
PSQI	RCTs	Serious	No	No	No	No	434	700	MD -1.38 (-2.45 to -0.31)	Low
ISI	RCTs	Serious	No	No	No	No	84	84	MD -1.11 (-3.68 to 1.45)	Low
HASD-A	RCTs	Serious	No	No	Serious	No	155	157	MD -1.24 (-2.41 to -0.35)	Moderate
HASD-D	RCTs	Serious	No	No	Serious	Serious	155	157	MD -1.18 (-2.15 to -0.20)	Moderate
BFI	RCTs	Serious	No	No	No	Serious	147	120	MD -2.3 (-5.62 to -1.03)	Low

Acupuncture versus control group.

No., Number; RCTs, randomized controlled trials; CI, confidence interval; MD, mean difference; PSQI, Pittsburgh Sleep Quality Index.

GRADE, Grading of Recommendation, Assessment, Development and Evaluation.

### Primary outcomes

#### The PSQI

**Figure 4:**
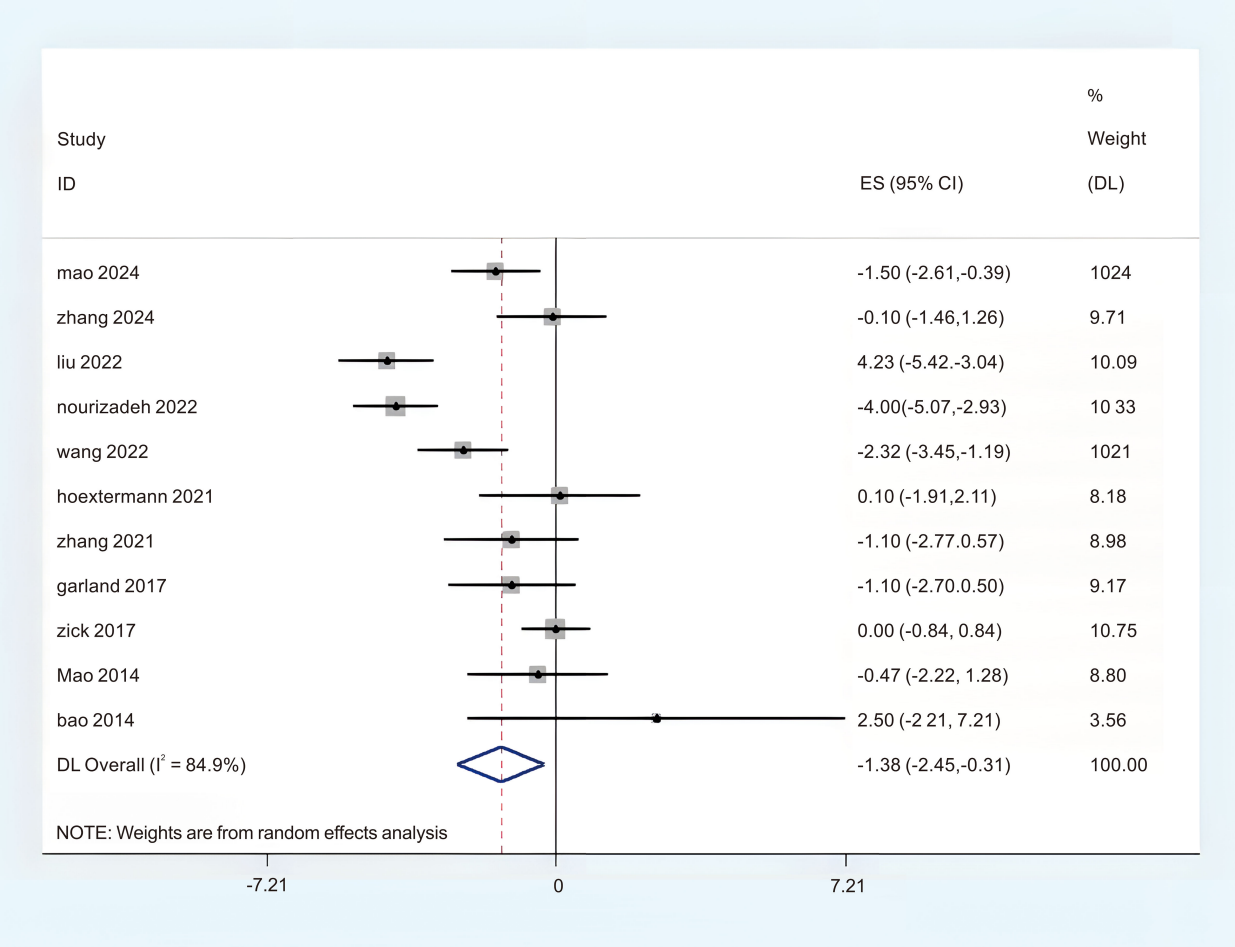
Forest plot of PSQI.

**Figure 5:**
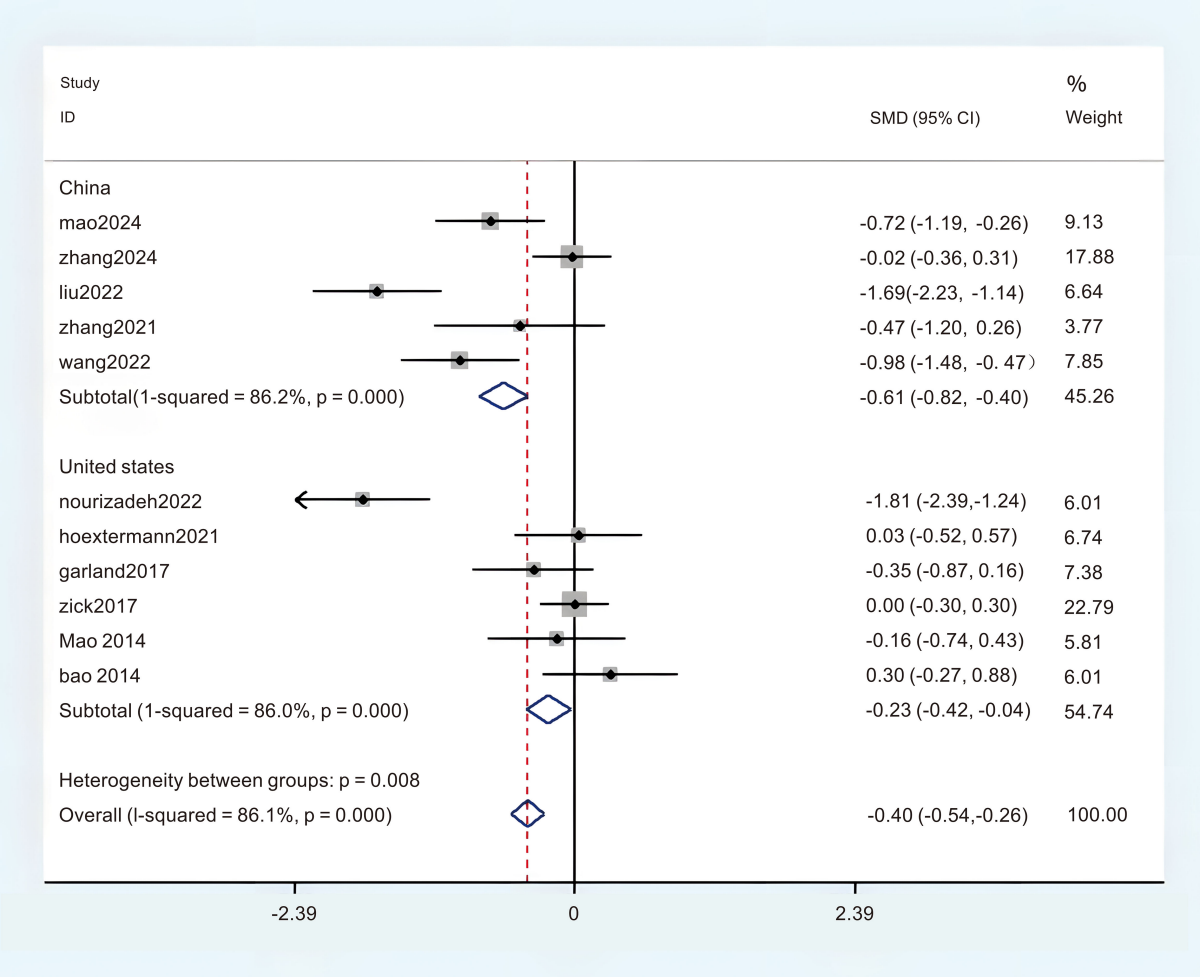
Subgroup analysis of PSQI in different countries.

**Figure 6:**
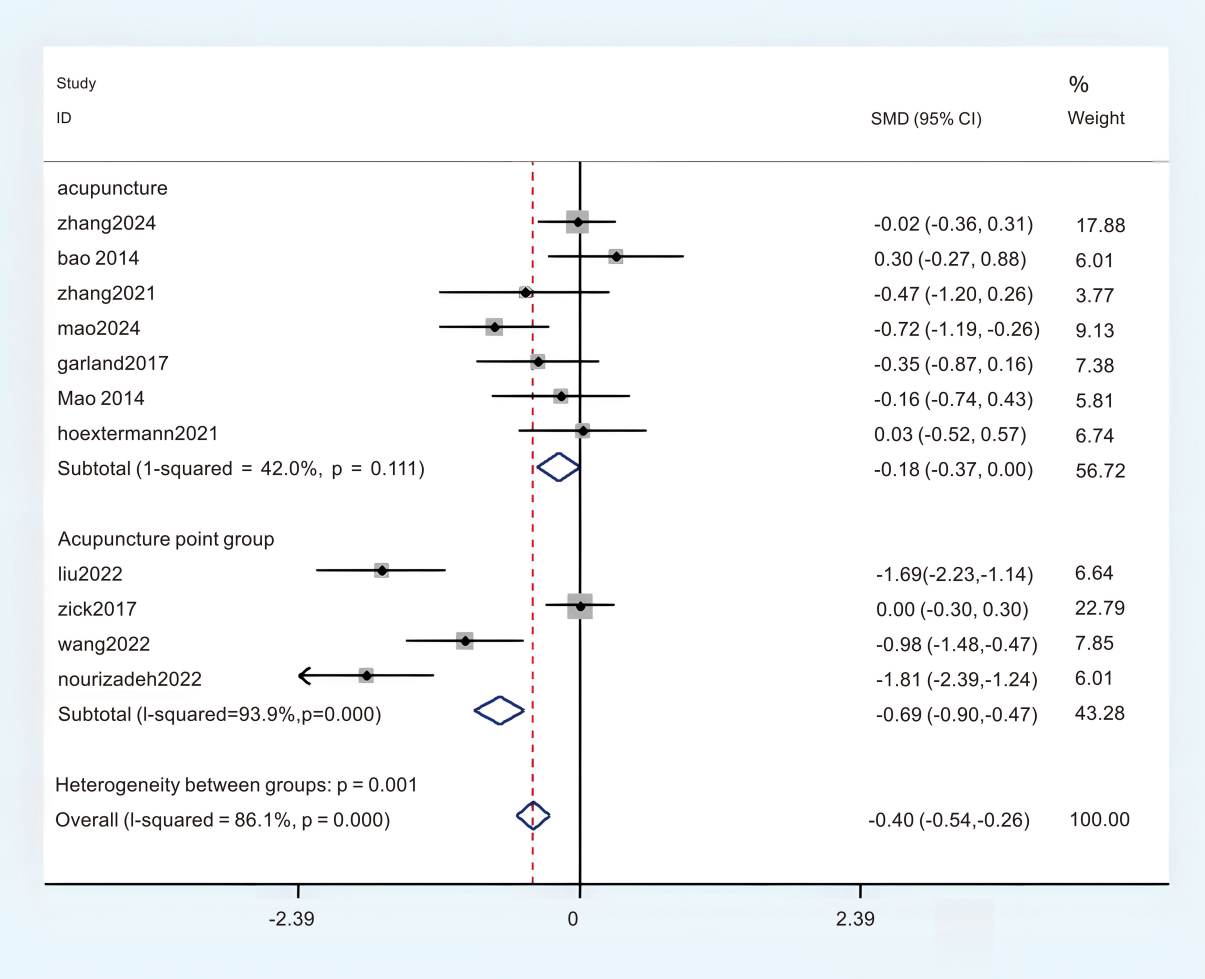
Subgroup analysis of PSQI in different intervention mode.

A total of 11 studies provided adequate data on the Pittsburgh Sleep Quality Index (PSQI), which were obtained in the meta-analysis. There was a notable degree of heterogeneity observed, leading to the selection of the REM (P < 0.1, I^2^ = 84.9%). The findings from the random effects meta-analysis indicated that acupuncture demonstrated a more pronounced effect on enhancing sleep quality compared to the experimental comparison group. (MD = -1.38; 95%CI = -2.45 to -0.31; **Figure 4**). To identify the sources of heterogeneity and assess the correlation of the Pittsburgh Sleep Quality Index (PSQI) across different countries, we categorized the PSQI results into two subgroups based on these nations. There were five studies conducted in China that provided data on the Pittsburgh Sleep Quality Index (PSQI), revealing a notable variability in the aggregated outcomes (P < 0.05, I^2^ = 86.2%), and six studies provided the Pittsburgh Sleep Quality Index (PSQI) data from the United States, revealing a notable variability in the aggregated outcomes (P < 0.05, I^2^ = 86.0%). In this subgroup analysis, a random effects model (REM) was employed as the statistical method. The results of the synthesis across subgroups are presented as follows: (1) in China (MD = -0.61; 95% CI = -0.82 to -0.40; **Figure 5**); (2) in Unite states (MD = -0.23; 95% CI = -0.29 to 0.25; **Figure 5**); In order to clarify the sources of heterogeneity and assess the relationship between PSQI and intervention models, we categorized the PSQI results into two subgroups based on two distinct intervention models. There were seven studies that utilized the Pittsburgh Sleep Quality Index (PSQI) in acupuncture research, revealing no substantial heterogeneity present within the aggregated data (P < 0.05, I^2^ = 42.0%). The Finite Element Method (FEM) was employed in the analysis of this subgroup. Four studies administered the Pittsburgh Sleep Quality Index (PSQI) in relation to acupressure, revealing a significant heterogeneity among the pooled data (P < 0.05, I^2^ = 93.9%), The REM was employed for this subgroup analysis. The results of the synthesis across subgroups are presented as follows: (1) acupuncture (MD = -0.18; 95% CI = -0.37 to 0.00; **Figure 6**); (2) acupressure (MD = -0.69; 95% CI = -0.90 to -0.47; **Figure 6**). The overall certainty of this evidence was assessed to be moderate to very low.

#### The BFI

**Figure 7:**
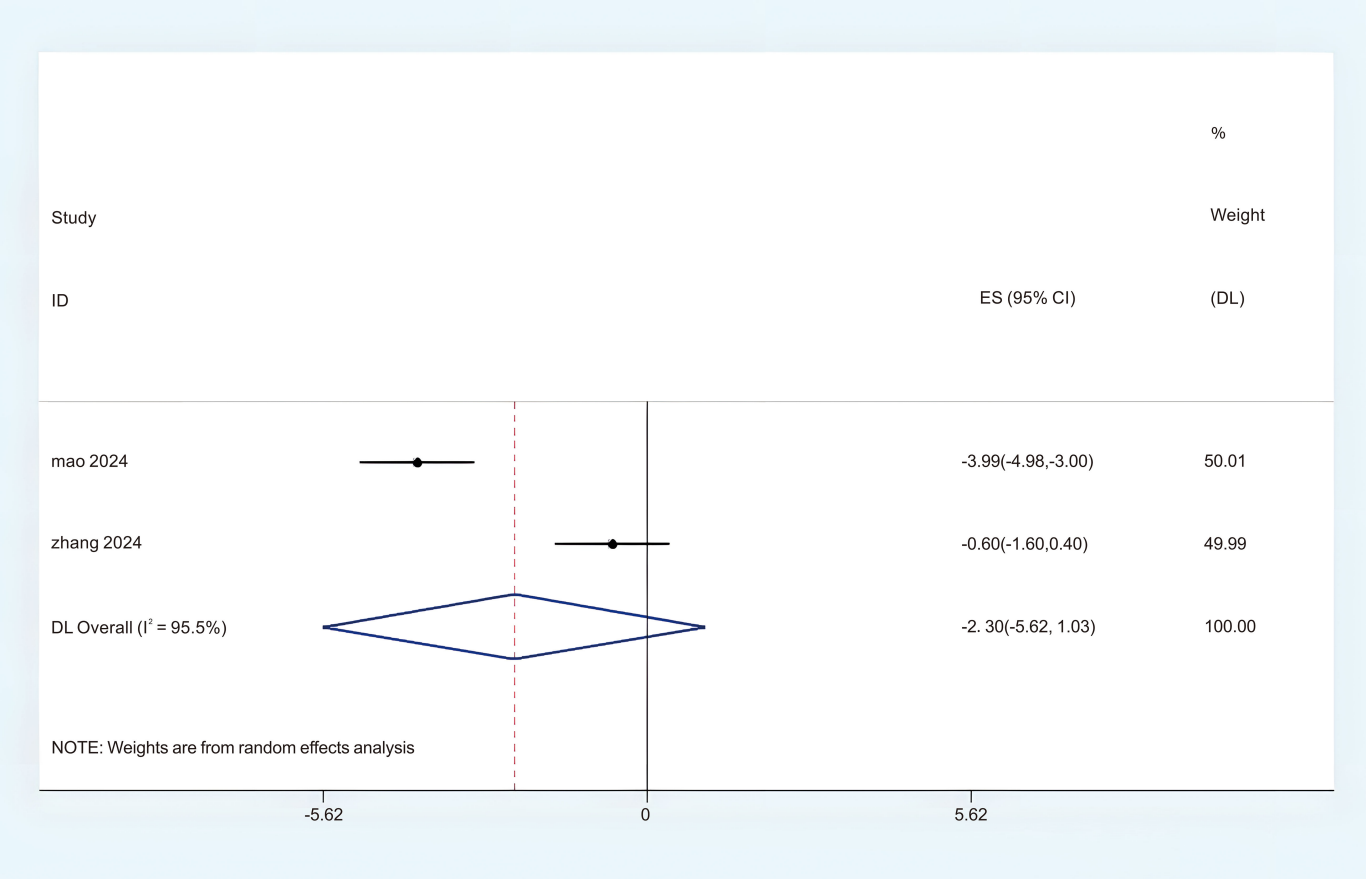
Forest plot of BFI.

**Figure 8:**
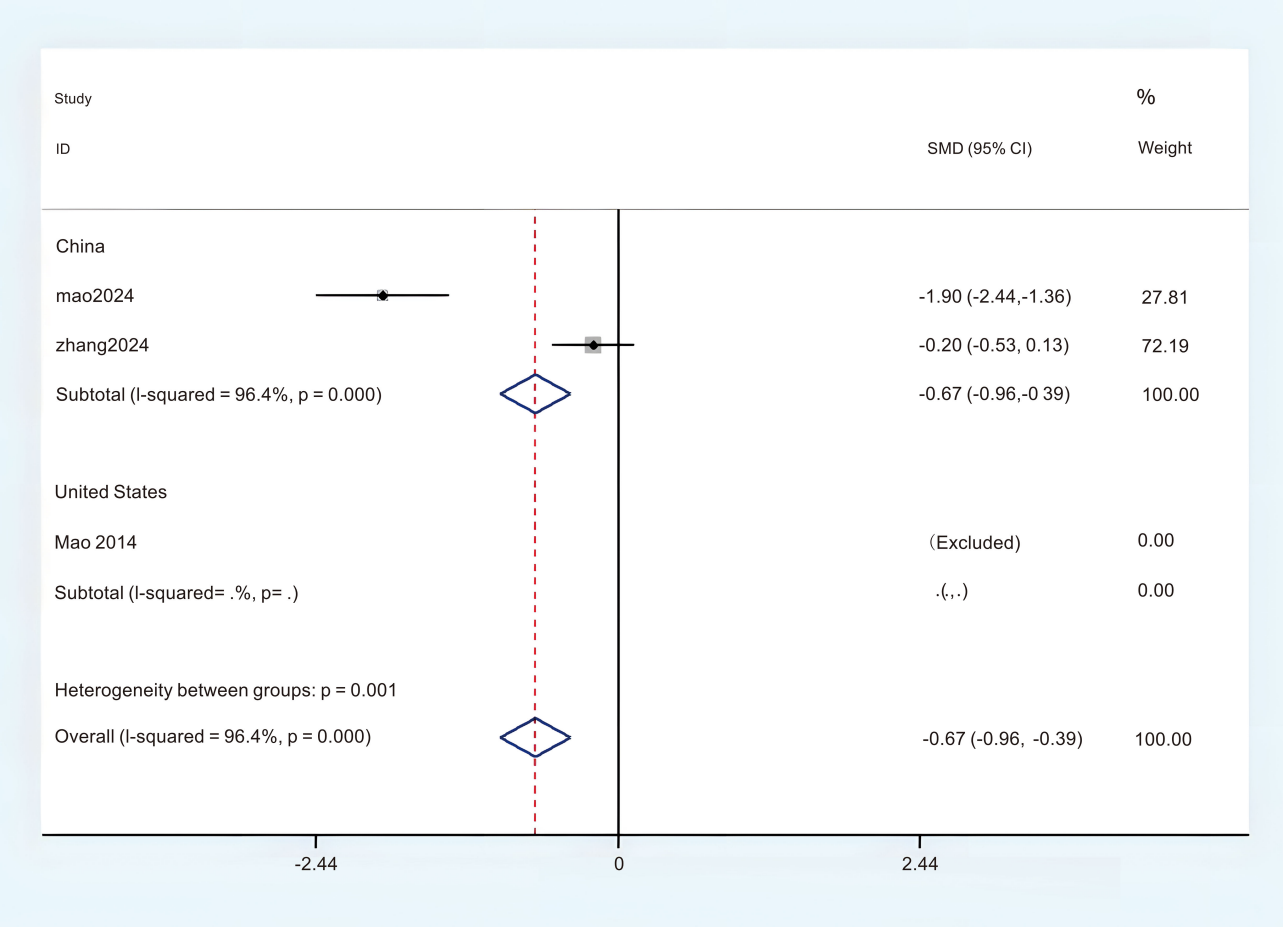
Subgroup analysis of BFI in different countries.

Three studies provided sufficient data regarding BFI for this meta-analysis. Based on the observed significant heterogeneity, a random effects model (REM) was determined to be appropriate (P < 0.05, I^2^ = 95.5%). The findings from the random effects meta-analysis indicated that acupuncture demonstrated a significantly greater impact on the enhancement of BFI compared to the control group. (MD = -2.30; 95%CI = -5.62 to -1.03; **Figure 7**). In order to investigate the sources of heterogeneity and assess the relationship of BFI across different countries, we classified the BFI results into two subgroups based on the circumstances of these two nations. There were two studies that administered the BFI in China, revealing a notable variability in the aggregated outcomes (P < 0.05, I^2^ = 96.4%). In this analysis, a random effects model (REM) was employed. The results of the subgroup synthesis are presented as follows: (1) in China (MD = -0.67; 95% CI = -0.96 to -0.39; **Figure 8**). The overall certainty of this evidence was assessed to be moderate to very low.

#### The ISI

**Figure 9:**
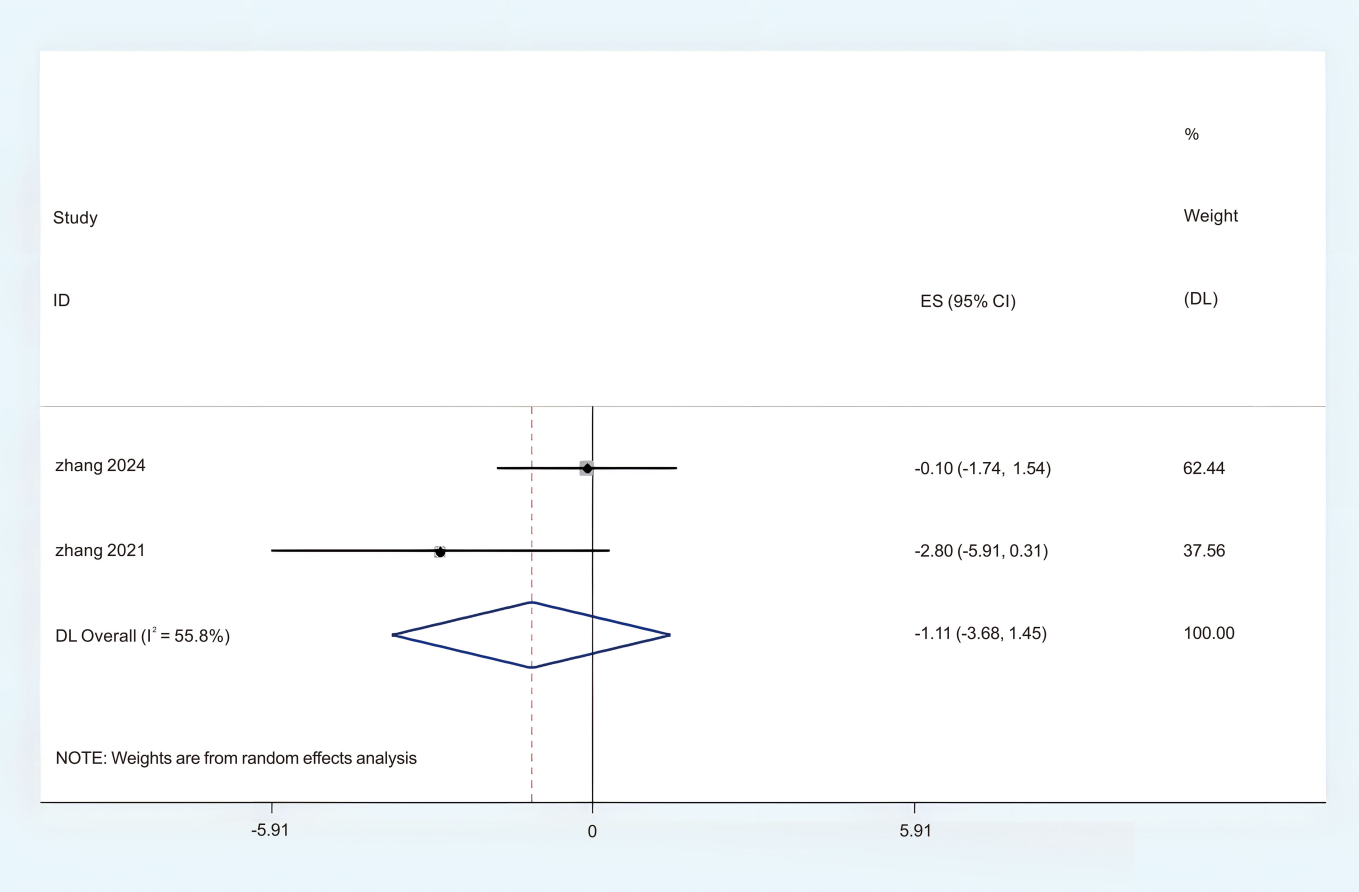
Forest plot of ISI.

**Figure 10:**
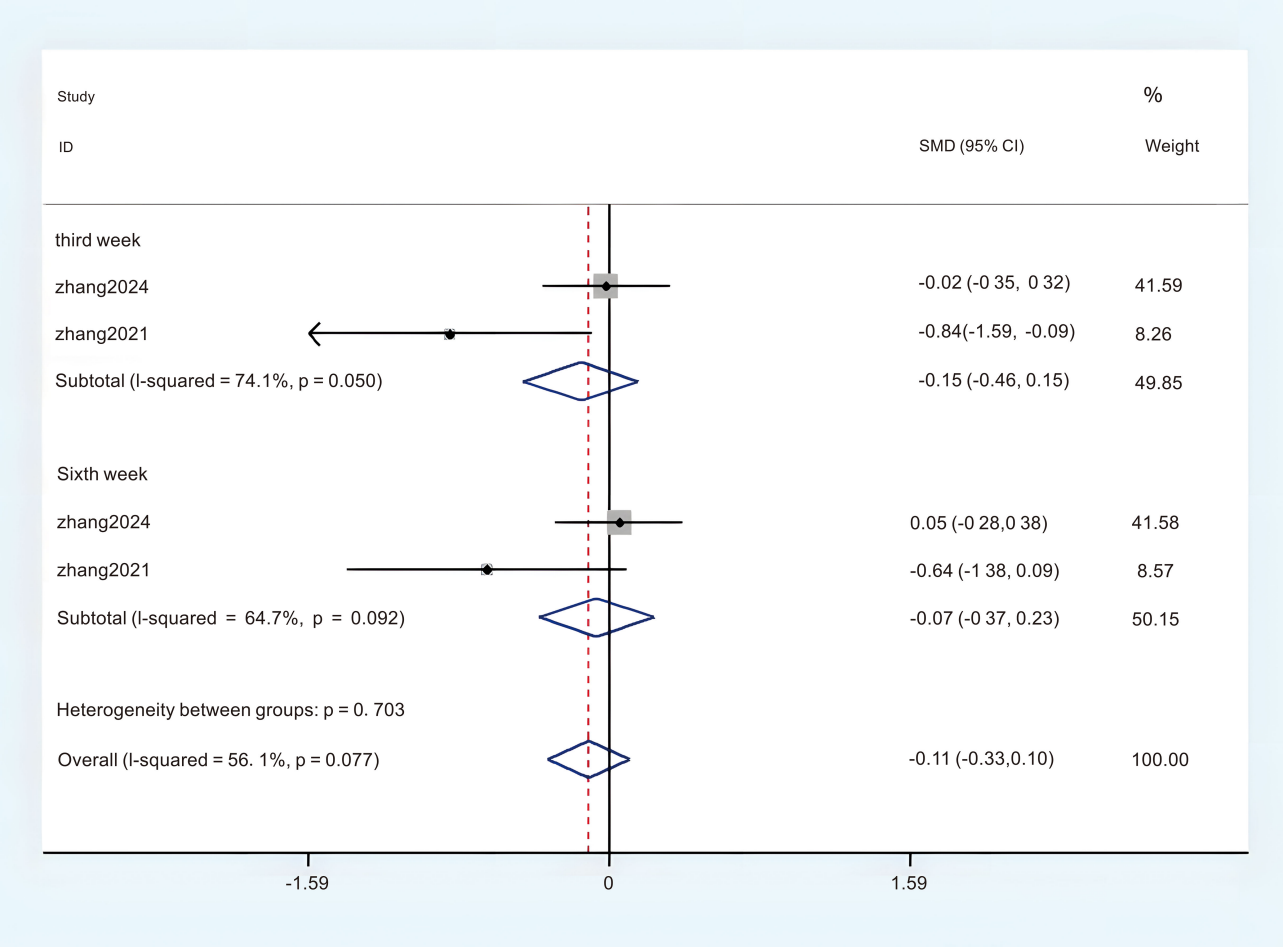
Subgroup analysis of ISI in different intervention sessions.

Two studies provided sufficient ISI data, which can be incorporated into this meta-analysis. Significant heterogeneity was observed, prompting the selection of a Random Effects Model (REM) (P < 0.05, I^2^ = 55.8%). The results of the meta-analysis given by random effects showed that acupuncture had a more significant effect on the improvement of ISI than the control group. (MD = -1.11; 95%CI = -3.68 to 1.45; **Figure 9**). Subgroup analyses were conducted to investigate potential sources of heterogeneity and examine the relationship between ISI scores and intervention sessions. The results stratified by intervention session duration demonstrated: (1) third week (MD = -0.15; 95% CI = -0.46 to -0.15; **Figure 10**); (2) sixth week (MD = -0.07; 95% CI = -0.37 to -0.23; **Figure 10**). The overall certainty of this evidence was assessed to be moderate to very low.

#### The HADS-A

**Figure 11:**
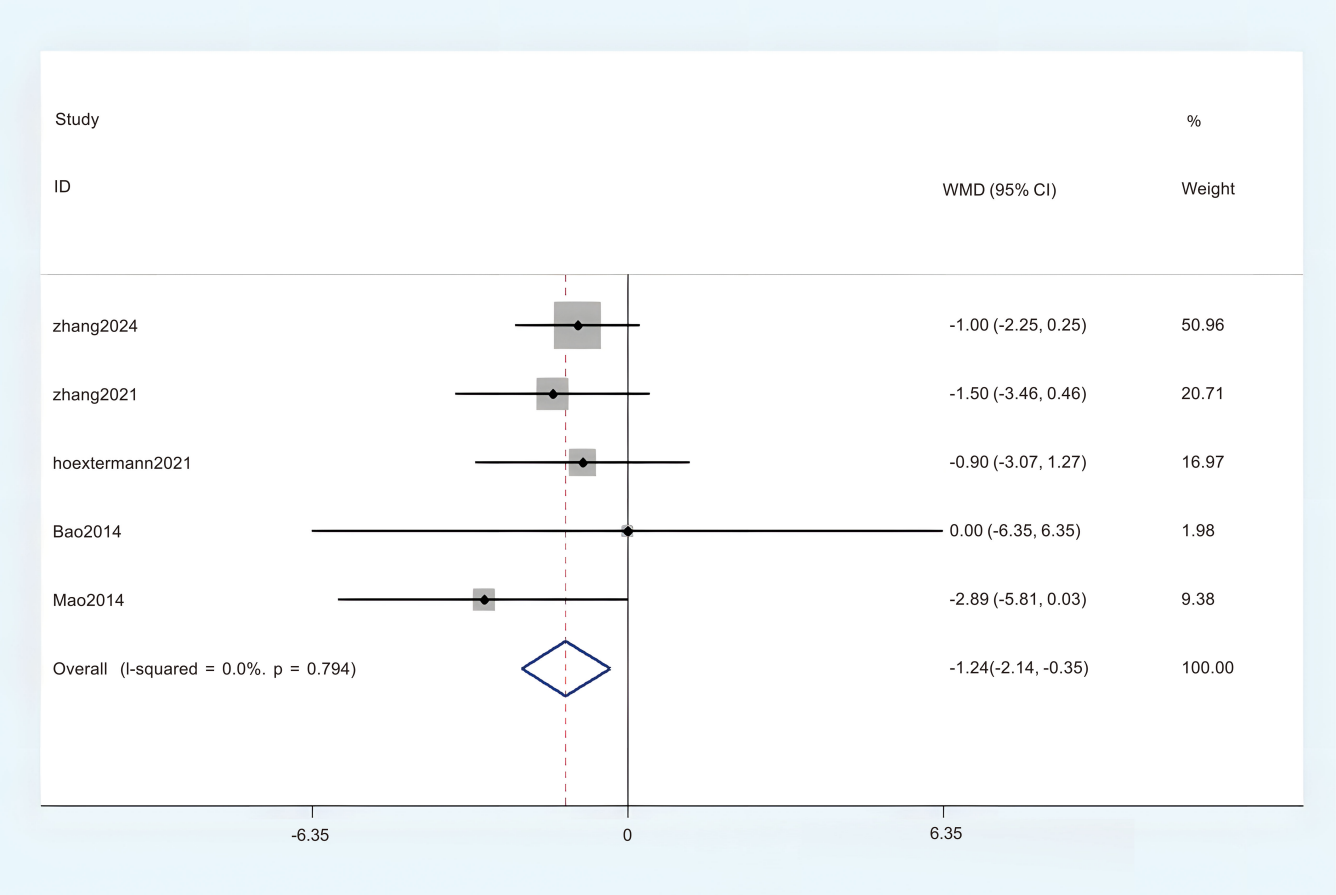
Forest plot of HADS-A.

Five studies provided sufficient data on the Hospital Anxiety and Depression Scale - Anxiety subscale (HADS-A) for this meta-analysis. No significant heterogeneity was observed, and a fixed-effects model (FEM) was employed (P < 0.1, I^2^ = 0%). The aggregated results revealed a statistically significant difference between the acupuncture group and the comparison group. (MD = -1.24; 95% CI = -2.41 to -0.35; **Figure 11**). The overall certainty of this evidence was assessed to be moderate to very low.

#### The HADS-D

**Figure 12:**
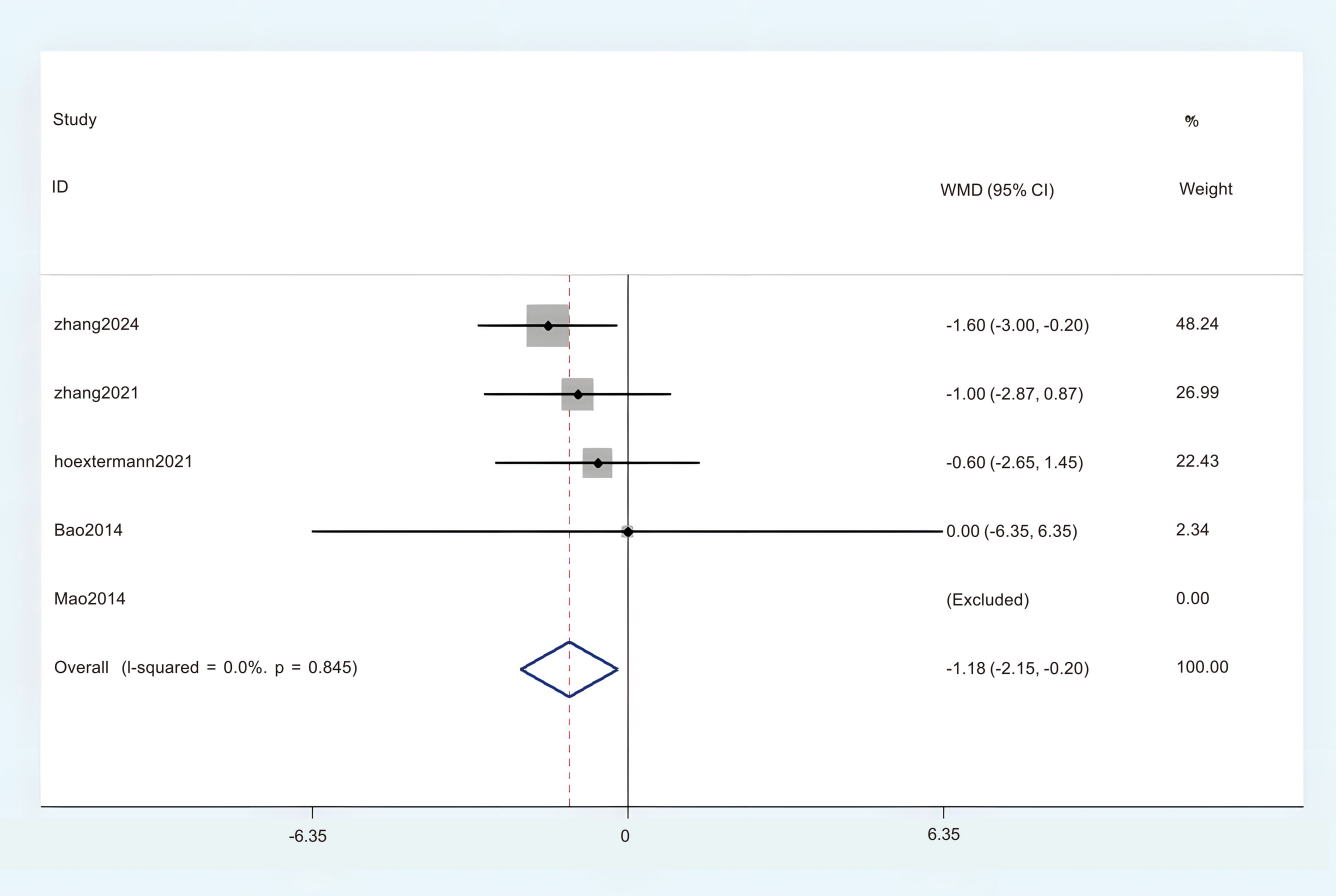
Forest plot of HADS-D.

A total of five studies contributed sufficient data on the Hospital Anxiety and Depression Scale-Depression (HADS-D) for this meta-analysis. The analysis revealed no significant heterogeneity, leading to the utilization of a Fixed Effects Model (FEM). (P < 0.1, I^2^ = 0%). The aggregated findings revealed a statistically significant difference between the acupuncture group and the control group. (MD = -1.18; 95% CI = -2.15 to -0.2; **Figure 12**). The overall certainty of this evidence was assessed to be moderate to very low.

**Figure 13:**
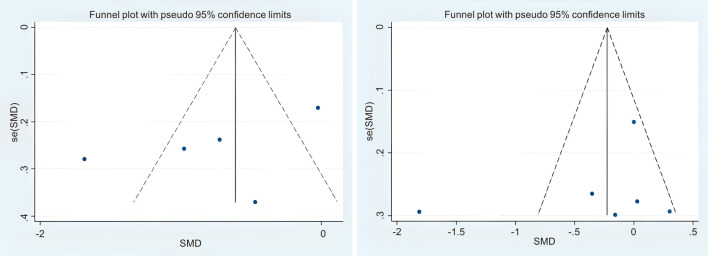
Funnel plot of PSQI.

Publication bias the funnel plot method was employed to assess publication bias (**Figure 13**), which indicated that there was no evidence of publication bias in the study.

## Discussion

The study conducted by Thompson et al (38) indicates that sleep disorders can lead to an increase in the secretion of inflammatory biomarkers, specifically including tumor necrosis factor-alpha (TNF-α), interleukin-6 (IL-6), and C-reactive protein (CRP). This is concomitant with a decrease in cortisol levels within the body. According to the traditional Chinese medicine, sleep quality disorders and emotional maladjustment are primarily attributed to imbalances in qi and blood, and they are also associated with the dysfunction of internal organs (39). Sleep disturbances and mood disorder, a typical and chronic symptom of breast cancer patients, can cause patient discomfort and diminish life. According to modern medicine, sleep disorders are caused by the excitement of autonomic nervous system. Therefore, research has demonstrated that acupuncture can enhance the treatment of sleep disorders and emotional disorder by stimulating the body’s parasympathetic nervous system (40). Relevant studies have indicated that inadequate sleep is linked to the use of sleep aids, anxiety disorders, and metastatic diseases (41). Acupuncture plays an important role as a traditional medical treatment, but its impact on research on the treatment of insomnia and emotional maladjustment in patients with breast cancer is controversial. Moreover, relevant studies have not clearly stated that acupuncture plays a good role in the treatment of breast cancer patients (19). Nighttime light exposure (such as staying up late or working night shifts) inhibits melatonin secretion, which has antioxidant effects, inhibits estrogen synthesis and regulates immunity. Its reduction may promote tumorigenesis. Although acupuncture can promote the stimulation of the body’s righteous qi, there remain numerous controversies regarding the management of sleep disorders and mood disorder in patients with breast cancer. In this study, we searched for randomized controlled trials of acupuncture to improve sleep quality and risk of emotional maladjustment of breast cancer patients abroad. In terms of improving sleep quality and enhancing the risk of emotional maladjustment for patients with breast cancer, acupuncture demonstrates significant differences when compared to other standard treatments.

Furthermore, subgroup analyses were conducted to enhance the validation of the findings from the study. We performed subgroup analyses based on various interventions and comparison group. The results indicated that acupuncture has a significant positive effect on improving the sleep quality of breast cancer patients when compared to standard care, pharmacological treatments, and sham compressions. Subgroup analyses were conducted for different countries and the results showed that acupuncture did considerably improve sleep quality in breast cancer patients compared with the control group. However, the subgroup analysis did not markedly reduce the heterogeneity present in the study. This discrepancy may arise from the variations in measurement methods employed between the intervention and control groups. In the study of the control group, it also played a therapeutic role, and this result is likely to be related to the psychological comfort given by medical staff (42). Therefore, there is an urgent need to conduct more high-quality randomized controlled trials in the future to compare the correlations between various intervention regimens and their respective effects. Additionally, these studies should aim to optimize the dose-response relationship to provide more substantial evidence supporting the research findings. For the ISI, BFI, the pooled results of our study indicated a high degree of heterogeneity between the acupuncture and control groups in the treatment of patients with insomnia disorder related to breast cancer. Therefore, we performed subgroup analyses but were unable to explore sources of heterogeneity, possibly due to inconsistencies in sample characteristics and intervention regimens across studies. Considering the limitations of existing research in both qualitative and quantitative aspects, as well as the potential influence of confounding factors, it is advisable to exercise caution when interpreting these subgroup results. For the included literature, there is bias in the quality of the currently included studies, which may lead to low quality of the research evidence, so it is suggested that the clinical design of acupuncture and moxibustion research should be standardized. In terms of outcome measures, no more comprehensive indicators were considered to comprehensively assess the overall efficacy of acupuncture therapy for breast cancer treatment. For the HADS-A, HDAS-D, the pooled results of our study indicated that no significant differences were observed between the acupuncture group and the control group in the treatment of emotional dysregulation associated with breast cancer. It is essential to recognize that the standard for selection and positioning of acupuncture in the studies included were not consistent. The accuracy of acupuncture localization has been recognized as a crucial determinant in achieving therapeutic efficacy. Future research adopt the standard acupuncture point chart of China to enhance the accuracy of locating and identifying acupuncture points. Descriptive analyses of objective sleep parameters have shown varying results across different studies. These discrepancies may arise from the differences in precision and sensitivity among various devices used to measure these parameters, potentially leading to inconsistent findings and introducing biases. Repetitive testing will be essential in the future to ensure further validation. Modifications in physiological biomarkers are imperative to furnish scientifically credible evidence. Consequently, it is imperative that future research endeavors account for these variables to the greatest extent feasible. Wang et al (43) showed that the application of acupuncture has been shown to enhance sleep quality and mood regulation by reducing heart rate and increasing heart rate variability; However, None of the studies included in this review examined these physiological parameters; therefore, additional evidence is required to establish a connection between alterations in physiological parameters and sleep promotion. In the majority of studies included in this review, the questionnaire survey serves as a tool for measuring outcomes. Given the subjective nature of sleep quality and mood regulation, patient reports play a crucial role in assessing prognosis, monitoring and managing symptoms, as well as guiding clinical decision-making. coincidentally, questionnaires are regarded as a cost-effective and easily accessible method, which contributes to their widespread application in clinical practice. However, by measuring sleep quality and risk of emotional maladjustment, We can minimize subjective biases from participants and evaluators, thereby gaining a deeper understanding of the changes in sleep architecture and circadian rhythms. In the future, a combination of subjective and objective assessment tools can be employed to evaluate sleep quality and risk of emotional maladjustment, facilitating the analysis of the correlation between objective monitoring data and patient-reported outcomes.

This meta-analysis also had a few limitations which should be stated. We conducted a comprehensive search of foreign databases and identified the absence of current meta-analyses examining the efficacy of acupuncture in enhancing sleep quality and risk of emotional maladjustment among patients diagnosed with breast cancer. Although there are many meta-sections on acupuncture to improve breast cancer, none of them involve the clinical heterogeneity analysis of the sleep quality and the risk of emotional maladjustment of patients with breast cancer, and no reasonable subgroup analysis is conducted, which has limited clinical guidance value. The meta-analysis of this paper summarized the latest evidence, analyzed the effect of acupuncture on the improvement of the sleep and emotion patients with breast cancer from the perspective of the largest sleep quality disorder and the risk of emotional maladjustment affecting breast cancer patients, and carried out a reasonable clinical heterogeneity and subgroup analysis. Only 11 articles were included in this study, with small sample size, low quality, large heterogeneity, low quality evidence for the results, and lack of evaluation of endpoint indicators. This article did not search Chinese databases, and the external validity conclusion was poor.

## Conclusion

The efficacy of acupuncture in enhancing sleep quality and risk of emotional maladjustment among breast cancer patients was significantly distinct from that of other standard treatments. However, because of the risk of bias and low quality of the evidence in the primary literature included in this study, the specific facts should be considered when applying it clinically.

## Data Availability

The original contributions presented in the study are included in the article/**Supplementary Material**. Further inquiries can be directed to the corresponding authors.

## References

[1] Giron PS Haddad CA Lopes de Almeida Rizzi SK Nazário AC Facina G . Effectiveness of acupuncture in rehabilitation of physical and functional disorders of women undergoing breast cancer surgery. Supportive Care Cancer. (2016) 24(6):2491–6. doi: 10.1007/s00520-015-3054-5 26670916

[2] Sung H Ferlay J Siegel RL Laversanne M Soerjomataram I Jemal A . Global cancer statistics 2020:GLOBAL estimates of incidence and mortality worldwide for 36 cancers in 185 countries. Ca-Cancer J Clin. (2021) 71:5. doi: 10.3322/caac.21660 33538338

[3] Siegel RL Miller KD Fuchs HE Jemal A . Cancer statistics. Ca-Cancer J Clin. (2022) 72:7–33. doi: 10.3322/caac.21708 35020204

[4] Wu HS Gao F Given C . Living as a survivor: sleep disturbance, fatigue, depressive mood, and cognitive dysfunction after breast cancer treatment. Cancer Nurs. (2024) 47(3):221-8. doi: 10.1097/NCC.0000000000001200 PMC10349906 36728181

[5] Cheng WH Teo RH Cheng LJ Lau Y Lau ST . Global prevalence of sleep disturbances among breast cancer survivors: a systematic review with metaanalysis. Sleep Health. (2023) 9:704–16. doi: 10.1016/j.sleh.2023.04.004 37453906

[6] Schroeck JL Ford J Conway EL Kurtzhalts KE Gee ME Vollmer KA . Review of safety and efficacy of sleep medicines in older adults, Clin. Therapeut. (2016) 38:2340–72. doi: 10.1016/j.clinthera.2016.09.010 27751669

[7] Lu WL Li HX Qian BY Wang Y Jansen L Huang GW . The clinical characteristics and prognosis of Chinese early stage breast cancer patients:a retrospective study. Breast J. (2010) 16(3):331. doi: 10.1111/j.1524-4741.2010.00903.x 20210801

[8] Murphy CC Fullington HM Alvarez CA Betts AC Lee S Haggstrom DA . Polypharmacy and patterns of prescription medication use among cancer survivors. Cancer. (2018) 124:2850–7. doi: 10.1002/cncr.31389 PMC6147245 29645083

[9] Ma Y Hall DL Ngo LH Liu Q Bain PA Yeh GY . Efficacy of cognitive behavioral therapy for insomnia in breast cancer: a meta-analysis, Sleep Med. Rev. (2021) 55:101376. doi: 10.1016/j.smrv.2020.101376 PMC8210529 32987319

[10] Yang H Yang Z Pan H Zhou Q . Effects of physical activity on sleep problems in breast cancer survivors: a meta-analysis, Support. Care Cancer. (2021) 29:4023–32. doi: 10.1007/s00520-020-05914-y 33403402

[11] Zhang J Qin Z So TH Chang TY Yang S Chen H . Acupuncture for chemotherapy-associated insomnia in breast cancer patients: an assessor participant blinded, randomized, sham-controlled trial. Breast Cancer Res. (2023) 25:49. doi: 10.1186/s13058-023-01645-0 37101228 PMC10134666

[12] Yang P Hu Q Zhang L Shen A Zhang Z Wang Q . Effects of non-pharmacological interventions on cancer-related cognitive impairment in patients with breast cancer: A systematic review and network meta-analysis. Eur J Oncol Nurs. (2025) 75:102804. doi: 10.1016/j.ejon.2025.102804 39919600

[13] Zhu J Chen X Zhen X Zheng H Chen H Chen H . Meta-analysis of effects of yoga exercise intervention on sleep quality in breast cancer patients, Front. Oncol. (2023) 13:1146433. doi: 10.3389/fonc.2023.1146433 PMC10348890 37456259

[14] Schapira MM Mackenzie ER Lam R Casarett D Seluzicki CM Barg FK . Breast cancer survivors willingness to participate in an acupuncture clinical trial: a qualitative study. Support Care Cancer. (2014) 22:1207–15. doi: 10.1007/s00520-013-2073-3 [DOI] [PMC free article] [PubMed] [Google Scholar PMC4162629 24362843

[15] Joshi N Araque H . Neurophysiologic basis for the relief of human pain by acupuncture. Acupunct Electrother Res. (2009) 34:165–74. doi: 10.3727/036012909803861022 20344884

[16] Filshie J Bolton T Browne D Ashley S . Acupuncture and self acupuncture for long-term treatment of vasomotor symptoms in cancer patients—audit and treatment algorithm. Acupunct Med. (2005) 23:171–80. doi: 10.1136/aim.23.4.171 16430125

[17] Mao JJ Tan T Li SQ Meghani SH Glanz K Bruner D . Attitudes and barriers towards participation in an acupuncture trial among breast cancer patients: a survey study. BMC Complement Altern Med. (2014) 14:7. doi: 10.1186/1472-6882-14-7 24400734 PMC3893614

[18] Weng Y Ren X Zu Z Xiao L Chen M . Efficacy and safety of acupuncture for the treatment of insomnia in breast cancer patients: a systematic review and meta-analysis. Complementary therapies Med. (2024) 86:103087. doi: 10.1016/j.ctim.2024.103087 39299655

[19] Zhang J Zhang Z Huang S Qiu X Lao L Huang Y . Acupuncture for cancer-related insomnia: A systematic review and meta-analysis. Phytomedicine. (2022) 102:154160. doi: 10.1016/j.phymed.2022.154160 35636168

[20] Fu C Zhao N Liu Z Yuan LH Xie C Yang WJ . Acupuncture improves peri-menopausal insomnia: a randomized controlled trial. Sleep. (2017) 40(11):5. doi: 10.1093/sleep/zsx153 29029258

[21] Liu Y Feng H Liu W Mao H Mo Y Yin Y . Regulation action and nerve electrophysiology mechanism of acupuncture on arousal state in patients of primary insomnia. Zhongguo Zhen Jiu. (2017) 37(1):19–23. doi: 10.13703/j.0255-2930.2017.01.004 29231317

[22] Zhao H Liu C Ye H Shi K Yao Y Wang M . Advancements in the physiopathological study of acupuncture treatment for insomnia: A review. Med (Baltimore). (2024) 103:e38476. doi: 10.1097/MD.0000000000038476 PMC11466160 38941420

[23] Eriksen WT Singerman L Romero SAD Bussell J Barg FK Mao JJ . Understanding the use of acupuncture for insomnia among cancer survivors: A qualitative study. J Altern Complement Med. (2018) 24:962–7. doi: 10.1089/acm.2018.0156 PMC6157314 30247951

[24] Liberati A Altman DG Tetzlaff J Mulrow C Gøtzsche PC Ioannidis JP . The PRISMA statement for reporting systematic reviews and meta-analyses of studies that evaluate healthcare interventions: explanation and elaboration. Bmj. (2009) 339:b2700. doi: 10.1136/bmj.b2700 19622552 PMC2714672

[25] Shea BJ Reeves BC Wells G Thuku M Hamel C Moran J . AMSTAR 2: a critical appraisal tool for systematic reviews that include randomised or non-randomised studies of healthcare interventions, or both. Bmj. (2017) 358:j4008. doi: 10.1136/bmj.j4008 28935701 PMC5833365

[26] Higgins JP Altman DG Gøtzsche PC Jüni P Moher D Oxman AD . The Cochrane Collaboration’s tool for assessing risk of bias in randomised trials. Bmj. (2011) 343:d5928. doi: 10.1136/bmj.d5928 22008217 PMC3196245

[27] Mao H Jin M Xie L Mao N Shen X Chen J . Infrared laser moxibustion for cancer-related fatigue in breast cancer survivors: a randomized controlled trial. Breast Cancer Res. (2024) 26.1:80. doi: 10.1186/s13058-024-01838-1 38773552 PMC11110353

[28] Zhang J Qin Z So TH Chang TY Yang S Chen H . Acupuncture for chemotherapy-associated insomnia in breast cancer patients: an assessor-participant blinded, randomized, sham-controlled trial. Breast Cancer Res. (2023) 25.1:49. doi: 10.1186/s13058-023-01645-0 37101228 PMC10134666

[29] Liu Q Wang C Wang Y Xu W Zhan C Wu J . Mindfulness-based stress reduction with acupressure for sleep quality in breast cancer patients with insomnia undergoing chemotherapy: a randomized controlled trial. Eur J Oncol Nurs. (2022) 61:1022195. doi: 10.1016/j.ejon.2022.102219 36257214

[30] Wang Y Li X Ji X Wu J Li J Zheng W . Adaptive auricular point acupressure for sleep disturbance in women with breast cancer: a randomized controlled trial. Evidence-Based Complementary Altern Med. (2022) 2022:8637386. doi: 10.1155/2022/8637386 PMC9640241 36353150

[31] Nourizadeh R Khanipour S Zamiri RE Namin MS Khalili A Hakimi S . A comparison of the effects of self-acupressure and aerobic exercises on sleep disorders of breast cancer survivors: a controlled randomized clinical trial. Med Acupuncture. (2022) 34.2:131–6. doi: 10.1089/acu.2020.1515 PMC9057882 35509876

[32] Zhang J Qin Z So TH Chen H Lam WL Yam LL . Electroacupuncture plus auricular acupressure for chemotherapy-associated insomnia in breast cancer patients: a pilot randomized controlled trial. Integr Cancer therapies. (2021) 20:153473542110191035. doi: 10.1177/15347354211019103 PMC8161840 34036813

[33] Höxtermann MD Buner K Haller H Kohl W Dobos G Reinisch M . Efficacy and safety of auricular acupuncture for the treatment of insomnia in breast cancer survivors: a randomized controlled trial. Cancers. (2021) 13(16):4082. doi: 10.3390/cancers13164082 34439234 PMC8394534

[34] Garland SN Xie SX Li Q Seluzicki C Basal C Mao JJ . Comparative effectiveness of electro-acupuncture versus gabapentin for sleep disturbances in breast cancer survivors with hot flashes: a randomized trial. Menopause. (2017) 24.5:517–23. doi: 10.1097/GME.0000000000000779 PMC5403590 27875389

[35] Zick SM Sen A Wyatt GK Murphy SL Arnedt JT Harris RE . Investigation of 2 types of self-administered acupressure for persistent cancer-related fatigue in breast cancer survivors: a randomized clinical trial. JAMA Oncol. (2016) 2(11):1470–6. doi: 10.1001/jamaoncol.2016.1867 27388752

[36] Bao T Cai L Snyder C Betts K Tarpinian K Gould J . Patient-reported outcomes in women with breast cancer enrolled in a dual-center, double-blind, randomized controlled trial assessing the effect of acupuncture in reducing aromatase inhibitor-induced musculoskeletal symptoms. Cancer. (2014) 120(3):381–9. doi: 10.1002/cncr.28352 PMC3946917 24375332

[37] Mao JJ Farrar JT Bruner D Zee J Bowman M Seluzicki C . Electroacupuncture for fatigue, sleep, and psychological distress in breast cancer patients with aromatase inhibitor-related arthralgia: a randomized trial. Cancer. (2014) 120(23):3744–51. doi: 10.1002/cncr.28917 PMC4239308 25077452

[38] Thompson KI Chau M Lorenzetti MS Hill LD Fins AI Tartar JL . Acute sleep deprivation disrupts emotion, cognition, inflammation, and cortisol in young healthy adults, Front, Behav. Neurosci. (2022) 16:945661. doi: 10.3389/fnbeh.2022.945661 PMC9538963 36212194

[39] Yang T Wang S Zhang X Liu L Liu Y Zhang C . Efficacy of auricular acupressure in maintenance haemodialysis patients: a systematic review and meta- analysis. J Clin Nurs. (2022) 31:508–19. doi: 10.1111/jocn.15966 34268817

[40] Pei M Chen J Dong S Yang B Yang K Wei L . Auricular acupressure for insomnia in patients with maintenance hemodialysis: a systematic review and meta-analysis, Front. Psychiatr. (2021) 12:576050. doi: 10.3389/fpsyt.2021.576050 PMC8326797 34349673

[41] Faiz SA Knox AS Fellman B Jaumally BA Pacheco GN Das A . Sleep disturbances based on patient reported outcomes in patients with breast cancer. Sleep Breathing. (2024) 28(6):2491–500. doi: 10.1007/s11325-024-03150-w PMC11874875 39225722

[42] Wang T Tan JB Yao LQ Huilin C Zhao I Eliseeva S . Effects of somatic acupoint stimulation on anxiety and depression in cancer patients: an updated systematic review of randomized controlled trials, Compl. Ther Clin Pract. (2023) 51:101735. doi: 10.1016/j.ctcp.2023.101735 36812735

[43] Wang L Cheng W Sun Z Xu Y Cheng G Gaischek I . Ear acupressure, heart rate, and heart rate variability in patients with insomnia. Evid. base Compl. Altern Med. (2013) 2013:763631. doi: 10.1155/2013/763631 PMC3583049 23476702

