# Supplementary material for: Effects of acupuncture on improving sleep quality and the risk of emotional maladjustment of breast cancer patients: a systematic review and meta-analysis
Source: Front Oncol. 2025 Jun 26;15:1617818. doi: 10.3389/fonc.2025.1617818 (PMC12241161; doi:10.3389/fonc.2025.1617818)
Supplement: Supplementary file 1 [file DataSheet1.docx]

**GRADE Assessment.**

**Acupuncture versus control group**

| Outcomes | Study  Design | Risk of Bias | Inconsistency | Indirectness | Imprecision | Other  Considerations | No. of Participants | | Absolute Effect (95% CI) | Quality |
| --- | --- | --- | --- | --- | --- | --- | --- | --- | --- | --- |
|  | | | | | | | acupuncture | Control |  |  |
| PSQI | RCTs | Serious | No | No | No | No | 434 | 700 | MD -1.38 (-2.45 to -0.31) | Low |
| ISI | RCTs | Serious | No | No | No | No | 84 | 84 | MD -1.11 (-3.68 to 1.45) | Low |
| HASD-A | RCTs | Serious | No | No | Serious | No | 155 | 157 | MD -1.24 (-2.41 to -0.35) | Moderate |
| HASD-D | RCTs | Serious | No | No | Serious | Serious | 155 | 157 | MD -1.18 (-2.15 to -0.20) | Moderate |
| BFI | RCTs | Serious | No | No | No | Serious | 147 | 120 | MD -2.3 (-5.62 to -1.03) | Low |

Note: No.: Number; RCTs: randomized controlled trials; CI: confidence interval; MD: mean difference; PSQI: Pittsburgh Sleep Quality Index;

GRADE, Grading of Recommendation, Assessment, Development and Evaluation;
