# Supplementary material for: Effects of acupuncture on improving sleep quality and the risk of emotional maladjustment of breast cancer patients: a systematic review and meta-analysis
Source: Front Oncol. 2025 Jun 26;15:1617818. doi: 10.3389/fonc.2025.1617818 (PMC12241161; doi:10.3389/fonc.2025.1617818)
Supplement: Supplementary file 2 [file DataSheet2.docx]

**S2 Table. Comprehensive search strategies.**

PubMed

| Number | Search terms | Results |
| --- | --- | --- |
| #1 | "Breast Neoplasms"[Mesh] |  |
| #2 | ((((((((((((((((((((((((((((((((((((Breast Neoplasm[Title/Abstract]) OR (Neoplasm, Breast[Title/Abstract])) OR (Neoplasms, Breast[Title/Abstract])) OR (Breast Tumors[Title/Abstract])) OR (Breast Tumor[Title/Abstract])) OR (Tumor, Breast[Title/Abstract])) OR (Tumors, Breast[Title/Abstract])) OR (Breast Cancer[Title/Abstract])) OR (Cancer, Breast[Title/Abstract])) OR (Cancer of Breast[Title/Abstract])) OR (Cancer of the Breast[Title/Abstract])) OR (Malignant Neoplasm of Breast[Title/Abstract])) OR (Breast Malignant Neoplasm[Title/Abstract])) OR (Breast Malignant Neoplasms[Title/Abstract])) OR (Malignant Tumor of Breast[Title/Abstract])) OR (Breast Malignant Tumor[Title/Abstract])) OR (Breast Malignant Tumors[Title/Abstract])) OR (Mammary Cancer[Title/Abstract])) OR (Cancer, Mammary[Title/Abstract])) OR (Cancers, Mammary[Title/Abstract])) OR (Mammary Cancers[Title/Abstract])) OR (Mammary Neoplasms, Human[Title/Abstract])) OR (Human Mammary Neoplasm[Title/Abstract])) OR (Human Mammary Neoplasms[Title/Abstract])) OR (Neoplasm, Human Mammary[Title/Abstract])) OR (Neoplasms, Human Mammary[Title/Abstract])) OR (Mammary Neoplasm, Human[Title/Abstract])) OR (Breast Carcinoma[Title/Abstract])) OR (Breast Carcinomas[Title/Abstract])) OR (Carcinoma, Breast[Title/Abstract])) OR (Carcinomas, Breast[Title/Abstract])) OR (Mammary Carcinoma, Human[Title/Abstract])) OR (Carcinoma, Human Mammary[Title/Abstract])) OR (Carcinomas, Human Mammary[Title/Abstract])) OR (Human Mammary Carcinomas[Title/Abstract])) OR (Mammary Carcinomas, Human[Title/Abstract])) OR (Human Mammary Carcinoma[Title/Abstract]) |  |
| #3 | #1 OR #2 |  |
| #4 | "Acupuncture Therapy"[Mesh] |  |
| #5 | (((((((((Acupuncture Treatment[Title/Abstract]) OR (Acupuncture Treatments[Title/Abstract])) OR (Treatment, Acupuncture[Title/Abstract])) OR (Therapy, Acupuncture[Title/Abstract])) OR (Pharmacoacupuncture Treatment[Title/Abstract])) OR (Treatment, Pharmacoacupuncture[Title/Abstract])) OR (Pharmacoacupuncture Therapy[Title/Abstract])) OR (Therapy, Pharmacoacupuncture[Title/Abstract])) OR (Acupotomy[Title/Abstract])) OR (Acupotomies[Title/Abstract]) |  |
| #6 | #4 OR #5 |  |
| #7 | randomized controlled trial[Publication Type] OR  randomized[Title/Abstract] OR  placebo[Title/Abstract] |  |
| #8 | #3 AND #6 AND #7 | 18 |

Web of Science

| Number | Search terms | Results |
| --- | --- | --- |
| #1 | Breast Neoplasms (Topic) or Breast Neoplasm (Topic) or Neoplasm, Breast (Topic) or Neoplasms, Breast (Topic) or Breast Tumors (Topic) or Breast Tumor (Topic) or Tumor, Breast (Topic) or Tumors, Breast (Topic) or Breast Cancer (Topic) or Cancer, Breast (Topic) or Cancer of Breast (Topic) or Cancer of the Breast (Topic) or Malignant Neoplasm of Breast (Topic) or Breast Malignant Neoplasm (Topic) or Breast Malignant Neoplasms (Topic) or Malignant Tumor of Breast (Topic) or Breast Malignant Tumor (Topic) or Breast Malignant Tumors (Topic) or Mammary Cancer (Topic) or Cancer, Mammary (Topic) or Cancers, Mammary (Topic) or Mammary Cancers (Topic) or Mammary Neoplasms, Human (Topic) or Human Mammary Neoplasm (Topic) or Human Mammary Neoplasms (Topic) or Neoplasm, Human Mammary (Topic) or Neoplasms, Human Mammary (Topic) or Mammary Neoplasm, Human (Topic) or Breast Carcinoma (Topic) or Breast Carcinomas (Topic) or Carcinoma, Breast (Topic) or Carcinomas, Breast (Topic) or Mammary Carcinoma, Human (Topic) or Carcinoma, Human Mammary (Topic) or Carcinomas, Human Mammary (Topic) or Human Mammary Carcinomas (Topic) or Mammary Carcinomas, Human (Topic) or Human Mammary Carcinoma |  |
| #2 | Acupuncture Therapy (Topic) or Acupuncture Treatment (Topic) or Acupuncture Treatments (Topic) or Treatment, Acupuncture (Topic) or Therapy, Acupuncture (Topic) or Pharmacoacupuncture Treatment (Topic) or Treatment, Pharmacoacupuncture (Topic) or Pharmacoacupuncture Therapy (Topic) or Therapy, Pharmacoacupuncture (Topic) or Acupotomy (Topic) or Acupotomies (Topic) |  |
| #3 | randomized controlled trial (Topic) or randomized (Topic) or placebo (Topic) or random (Topic) or rct (Topic) |  |
| #4 | #1 AND#2 AND #3 | 526 |

Embase

| Number | Search terms | Results |
| --- | --- | --- |
| #1 | 'Breast Neoplasm':ab,ti or 'Neoplasm, Breast':ab,ti or 'Neoplasms, Breast':ab,ti or 'Breast Tumors':ab,ti or 'Breast Tumor':ab,ti or 'Tumor, Breast':ab,ti or 'Tumors, Breast':ab,ti or 'Breast Cancer':ab,ti or 'Cancer, Breast':ab,ti or 'Cancer of Breast':ab,ti or 'Cancer of the Breast':ab,ti or 'Malignant Neoplasm of Breast':ab,ti or 'Breast Malignant Neoplasm':ab,ti or 'Breast Malignant Neoplasms':ab,ti or 'Malignant Tumor of Breast':ab,ti or 'Breast Malignant Tumor':ab,ti or 'Breast Malignant Tumors':ab,ti or 'Mammary Cancer':ab,ti or 'Cancer, Mammary':ab,ti or 'Cancers, Mammary':ab,ti or 'Mammary Cancers':ab,ti or 'Mammary Neoplasms, Human':ab,ti or 'Human Mammary Neoplasm':ab,ti or 'Human Mammary Neoplasms':ab,ti or 'Neoplasm, Human Mammary':ab,ti or 'Neoplasms, Human Mammary':ab,ti or 'Mammary Neoplasm, Human':ab,ti or 'Breast Carcinoma':ab,ti or 'Breast Carcinomas':ab,ti or 'Carcinoma, Breast':ab,ti or 'Carcinomas, Breast':ab,ti or 'Mammary Carcinoma, Human':ab,ti or 'Carcinoma, Human Mammary':ab,ti or 'Carcinomas, Human Mammary':ab,ti or 'Human Mammary Carcinomas':ab,ti or 'Mammary Carcinomas, Human':ab,ti or 'Human Mammary Carcinoma':ab,ti |  |
| #2 | 'Acupuncture Treatment':ab,ti or 'Acupuncture Treatments':ab,ti or 'Treatment, Acupuncture':ab,ti or 'Therapy, Acupuncture':ab,ti or 'Pharmacoacupuncture Treatment':ab,ti or 'Treatment, Pharmacoacupuncture':ab,ti or 'Pharmacoacupuncture Therapy':ab,ti or 'Therapy, Pharmacoacupuncture':ab,ti or 'Acupotomy':ab,ti or 'Acupotomies':ab,ti |  |
| #3 | 'randomized controlled trial':ab,ti or 'randomized':ab,ti or 'placebo':ab,ti |  |
| #4 | #1 AND#2 AND #3 | 452 |
